# Chikungunya Cases Identified Through Passive Surveillance and Household Investigations — Puerto Rico, May 5–August 12, 2014

**Published:** 2014-12-05

**Authors:** Tyler M. Sharp, Nicole M. Roth, Jomil Torres, Kyle R. Ryff, Nicole M. Pérez Rodríguez, Chanis Mercado, Maria del Pilar Diaz Padró, Maria Ramos, Raina Phillips, Matthew Lozier, Carmen S. Arriola, Michael Johansson, Elizabeth Hunsperger, Jorge L. Muñoz-Jordán, Harold S. Margolis, Brenda Rivera García

**Affiliations:** 1 Division of Vector-Borne Diseases, National Center for Emerging and Zoonotic Infectious Diseases, CDC; 2 Office of Epidemiology, Puerto Rico Department of Health; 3 Division of Environmental Hazards and Health Effects, National Center for Environmental Health, CDC; 4 Epidemic Intelligence Service, CDC; 5 Influenza Division, National Center for Immunization and Respiratory Diseases, CDC; 6 Division of Global Health Protection, Center for Global Health, CDC

Chikungunya and dengue are mosquito-borne, viral, acute febrile illnesses that can be difficult to distinguish clinically. Whereas dengue is endemic in many countries in the Caribbean and the Americas, the first locally acquired chikungunya case in the Western Hemisphere was reported from the Caribbean island of St. Martin in December 2013 and was soon followed by cases in many parts of the region (1). In January 2014, the Puerto Rico Department of Health (PRDH) and CDC initiated chikungunya surveillance by building on an existing passive dengue surveillance system. To assess the extent of chikungunya in Puerto Rico, the severity of illnesses, and the health care–seeking behaviors of residents, PRDH and CDC analyzed data from passive surveillance and investigations conducted around the households of laboratory-positive chikungunya patients. Passive surveillance indicated that the first locally acquired, laboratory-positive chikungunya case in Puerto Rico was in a patient with illness onset on May 5, 2014. By August 12, a total of 10,201 suspected chikungunya cases (282 per 100,000 residents) had been reported. Specimens from 2,910 suspected cases were tested, and 1,975 (68%) were positive for chikungunya virus (CHIKV) infection. Four deaths were reported. The household investigations found that, of 250 participants, 70 (28%) tested positive for current or recent CHIKV infection, including 59 (84%) who reported illness within the preceding 3 months. Of 25 laboratory-positive participants that sought medical care, five (20%) were diagnosed with chikungunya and two (8%) were reported to PRDH. These investigative efforts indicated that chikungunya cases were underrecognized and underreported, prompting PRDH to conduct information campaigns to increase knowledge of the disease among health care professionals and the public. PRDH and CDC recommended that health care providers manage suspected chikungunya cases as they do dengue because of the similarities in symptoms and increased risk for complications in dengue patients that are not appropriately managed. Residents of and travelers to the tropics can minimize their risk for both chikungunya and dengue by taking standard measures to avoid mosquito bites.

Chikungunya* is an emerging infectious disease characterized by fever and arthralgia (2). After the first locally acquired chikungunya case was reported from St. Martin in December 2013, CHIKV spread rapidly throughout the Americas, with nearly 1 million cases reported to date.† Both CHIKV and the four dengue viruses (DENV-1–4) are transmitted by *Aedes aegypti* and *Aedes albopictus* mosquitoes. Dengue§ is endemic in Puerto Rico (3) and throughout the tropics¶ where these mosquitoes exist (4), and is characterized by fever, aches and pains, leukopenia, and minor bleeding manifestations (e.g., petechial and gingival bleeding) (5). Whereas DENV infection does not lead to long-lasting cross-protective immunity but rather is associated with increased risk for developing severe dengue after infection with another DENV, infection with CHIKV results in long-lived immunity that protects from future illness.

In January 2014, PRDH and CDC initiated chikungunya surveillance in Puerto Rico by modifying the existing Passive Dengue Surveillance System (PDSS) (3) to include suspected chikungunya. Patients for whom a clinician suspected chikungunya as the cause of illness were reported by sending a serum specimen along with a dengue case investigation form** on which “suspected chikungunya” was indicated. Specimens collected within 5 days of illness onset were tested by real-time reverse transcription–polymerase chain reaction (rRT-PCR) (6) with updated primers to detect current CHIKV infection. Specimens collected ≥6 days after illness onset were tested by immunoglobulin M capture enzyme-linked immunosorbent assay (MAC-ELISA) (7) to detect recent CHIKV infection. Specimens from suspected dengue patients and some specimens from suspected chikungunya patients were tested by rRT-PCR (8) or MAC ELISA to detect current or recent DENV infection, respectively. Laboratory-positive chikungunya cases were defined as suspected chikungunya cases with test results indicating current or recent CHIKV infection.

## Epidemiologic and Laboratory Investigation

### Passive surveillance analysis

**FIGURE 1:**
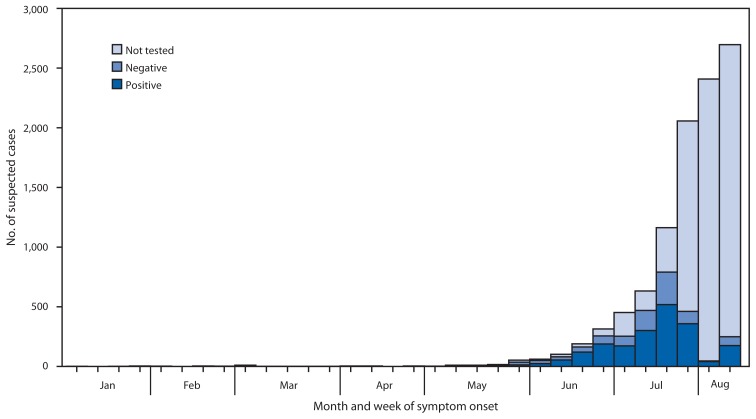
Week of symptom onset and testing status for suspected chikungunya cases reported to the Puerto Rico Department of Health — Puerto Rico, January 1–August 12, 2014

**FIGURE 2:**
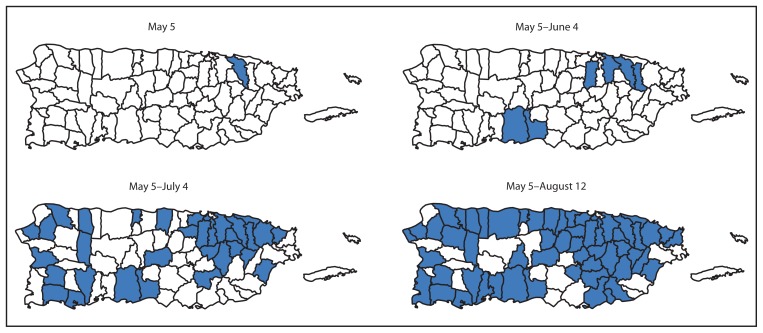
Geographic distribution of laboratory-positive chikungunya cases, by period and residence — Puerto Rico, May 5–August 12, 2014

**FIGURE 3:**
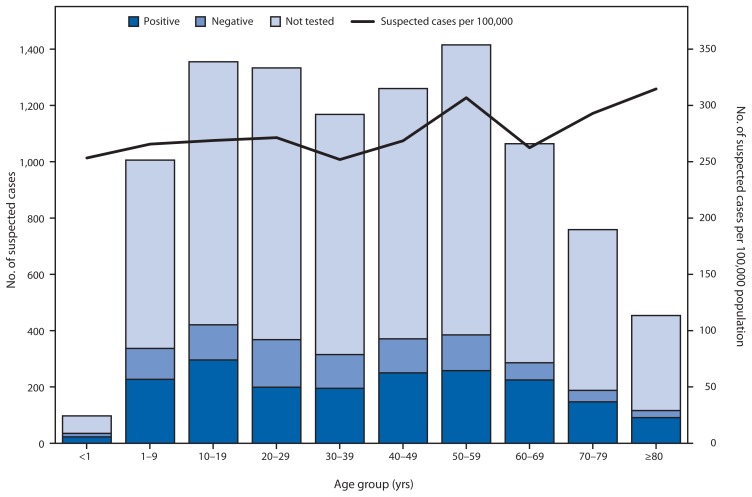
Number of suspected chikungunya cases, by age group* and test status, and number per 100,000 population — Puerto Rico, January 1–August 12, 2014 * Age was available for 9,911 suspected chikungunya cases.

Suspected chikungunya cases were first reported in January 2014, and the first laboratory-positive patient had illness onset on May 5 (Figure 1). The patient was a resident of the San Juan metropolitan area and did not report travel outside of Puerto Rico in the 14 days before illness onset. Additional laboratory-positive chikungunya cases were reported in the following weeks from throughout the San Juan and Ponce metropolitan areas (Figure 2). By August 12, a total of 10,201 suspected chikungunya cases (282 per 100,000 residents) had been reported from 57 (73%) of Puerto Rico’s 78 municipalities. Specimens from 2,910 suspected chikungunya cases were tested for CHIKV infection, and 1,975 (68%) were laboratory-positive. Suspected and laboratory-positive chikungunya cases were reported in all age groups, and incidence was highest among persons aged ≥50 years (291 suspected and 57 laboratory-positive cases per 100,000 residents) (Figure 3).

Of 652 laboratory-positive cases for which demographic and clinical information was available, 344 (53%) were in females, and the most commonly reported symptoms were fever (87%), arthralgia (79%), and myalgia (79%) (Table). Hospitalization was uncommon (13%), and major bleeding (3%) or severe manifestations (2%) were rarely reported.

Four deaths were reported among laboratory-positive chikungunya patients. The first patient was a woman aged 31 years with coexisting medical conditions (i.e., morbid obesity, diabetes, hypothyroidism, and asthma) who was brought to the emergency department because of difficulty breathing and acute febrile illness. She died within 24 hours of hospitalization. The second and third patients were men aged 45 and 78 years with preexisting diabetes. Both sought care for acute febrile illness, were diagnosed with a localized source of bacterial infection that was not confirmed by bacterial culture, and died <24 hours and 3 days after hospitalization, respectively. The fourth patient was a man aged 57 years with a history of congestive heart failure, diabetes, and obesity, who was found in his home unresponsive, febrile, and with labored breathing. A history of acute febrile illness was obtained, and an electrocardiogram revealed evidence of recent myocardial infarction. He died <24 hours after hospitalization.

### DENV and CHIKV cross-testing

Of 4,433 suspected dengue cases reported to PDSS during January 1–August 12, 426 (9.6%) were laboratory-positive for DENV infection. Of 147 suspected dengue cases that were laboratory-negative for DENV infection and subsequently tested for CHIKV infection, 21 (14%) were laboratory-positive. Of 761 suspected chikungunya cases also tested for DENV infection, 14 (2%) were laboratory-positive for DENV infection. Of 908 suspected dengue or chikungunya cases tested for infection with both viruses, none were concurrently infected.

### Household investigations

Household-based cluster investigations were conducted to 1) describe the spectrum of disease in CHIKV-infected persons; 2) determine the health care–seeking behaviors of persons with chikungunya; and 3) determine whether persons with chikungunya who sought medical care were appropriately diagnosed and reported. The rationale for the design of this investigation was based on prior observations that dengue cases cluster within households and neighborhoods (9) because of the movement of infected mosquitoes and humans (10), whereas human movement is the major vehicle for DENV dispersal >100 meters (11). Because the same pattern is presumed to be true for CHIKV transmission, additional infected persons were expected to be found among neighbors of chikungunya patients.

The residences of a convenience sample of reported laboratory-positive chikungunya patients were visited, and residents of households within a 50-meter radius were offered chikungunya diagnostic testing. Participants provided a serum specimen and answered a questionnaire regarding household characteristics, demographics, travel history, and recent illnesses. Laboratory-positive participants were household investigation participants with current or recent CHIKV infection.

During June 20–August 19, a total of 21 household investigations were conducted in the health regions of San Juan (nine investigations), Bayamón (eight), Ponce (two), Arecibo and Caguas (one each). Of 499 houses visited, 433 (87%) were occupied, and an adult was present at the time of visit at 200 (46%). From these 200 eligible households, a total of 250 residents in 137 (69%) of the households agreed to participate in the investigation. The median number of residents per participating household was three (range = 1–9), and a mean of two (range = 1–6) residents per household participated in the investigation. Median age of the participants (45 years) was higher than that of persons who did not participate in the investigation but lived in participating households (25 years).

Of the 250 household investigation participants, 70 (28%) were laboratory-positive for CHIKV infection, including 12 (17%) who had current and 58 (83%) who had recent CHIKV infection. Of the 70 who were laboratory-positive for CHIKV infection, 59 (84%) reported an acute illness in the preceding 3 months (Table). Of these 59, a total of 56 (95%) reported arthralgia, 55 (93%) reported fever, and 53 (90%) reported fever and arthralgia. Median duration of illness was 6 days (range = 2–21 days). After excluding the index patient for each household cluster, 25 (63%) of 40 laboratory-positive symptomatic participants sought medical care, of whom five (20%) were diagnosed with chikungunya, three (12%) were hospitalized, and two (8%) were reported to PRDH as having suspected chikungunya.

## Public Health Response

In accordance with International Health Regulations, CDC was notified after the first locally acquired chikungunya case in Puerto Rico was identified. The public health response to the impending epidemic focused on raising chikungunya awareness, both among health care providers and the public, and implementing syndromic surveillance with laboratory testing to enable timely detection of cases. Messaging to health care providers focused on the signs and symptoms of chikungunya and the need to manage suspected chikungunya patients as dengue patients because of their clinical similarity and the increased risk for morbidity and mortality if dengue patients are not managed appropriately (5,12). Mandatory reporting of novel diseases such as chikungunya was already required in Puerto Rico; however, supplemental regulations were issued requiring reporting of suspected chikungunya cases to PRDH. To enable more timely surveillance, a chikungunya sentinel surveillance system was initiated in August with nine representative health facilities selected to monitor epidemiologic trends.

### Discussion

CHIKV was first detected in modern-day Tanzania in 1952–1953 (13), and later caused outbreaks in countries in the Indian Ocean and southern Asia (2). Chikungunya outbreaks typically affect a large proportion of the population (e.g., 38%–63%) because of high viremia in the host and infected mosquitoes, inability to control vector mosquitoes, and lack of preexisting protective immunity (2). Some chikungunya patients experience prolonged morbidity and disability because of joint pain that can persist for months or years (14). Fatal chikungunya cases are rare (i.e., <0.1% of cases) and are typically associated with underlying health conditions or very young or advanced age (15). Although there is no specific treatment for either dengue or chikungunya, close clinical monitoring of dengue patients along with judicious fluid management can reduce morbidity and mortality (5,12).

Passive surveillance for any illness is dependent on ill persons seeking medical care, clinician recognition of the illness, and reporting of cases to public health authorities. The household investigations conducted during the chikungunya epidemic in Puerto Rico identified cases that had not been reported, suggesting that the magnitude of the epidemic is larger than suggested by passive surveillance. Because the population of Puerto Rico is presumed to be immunologically naïve with respect to CHIKV infection and *Ae. aegypti* mosquitoes are present year-round, the high infection rates observed in the household investigations were not unexpected. Prior investigations have reported rates of asymptomatic CHIKV infection of 3%–28% (16,17), similar to the rate observed in this investigation.

What is already known on this topic?

Chikungunya is an emerging infectious disease caused by chikungunya virus, which is transmitted via the bite of infected *Aedes aegypti* and *Aedes albopictus* mosquitoes and was introduced into the Western Hemisphere in late 2013. Because of clinical similarity with dengue, which is endemic throughout the tropics and depends on early identification and proper management to reduce morbidity and mortality, patients with suspected chikungunya should be managed according to recommended strategies for dengue patients.

What is added by this report?

The first locally acquired, laboratory-confirmed chikungunya case was detected in Puerto Rico in early May 2014, and 10,201 suspected cases (282 per 100,000 residents) had been reported by August 12. Fever and arthralgia were reported in most chikungunya patients, of whom 13% were hospitalized and four died. A series of household investigations found that, of 250 participants, 70 persons (28%) tested positive for current or recent chikungunya virus infection, including 59 who reported illness within the preceding 3 months. Among 25 participants with chikungunya that sought medical care, only two (8%) had been reported to health authorities. The identification of chikungunya patients through household investigations suggests that the actual incidence of chikungunya is higher than demonstrated by passive surveillance data.

What are the implications for public health practice?

Improved vigilance and reporting of suspected chikungunya cases by health care providers can help health authorities estimate the health burden of chikungunya in Puerto Rico and help mobilize the resources needed to respond to the epidemic and direct them to affected areas. The chikungunya epidemic is expected to continue until a critical threshold of the population is no longer susceptible to infection, until which time early and accurate identification of dengue patients will remain a challenge.

Because of overlapping signs and symptoms, chikungunya and dengue are often difficult to distinguish clinically. Therefore, surveillance in areas where both CHIKV and DENV are circulating should include laboratory diagnostic testing for both illnesses. Although aches and pains are characteristic of both illnesses, arthralgia might be more prominent in patients with chikungunya, whereas dengue patients typically complain of generalized myalgia and retro-orbital eye pain. Similarly, although rash might be present in both dengue and chikungunya patients, studies have suggested that it is more common and develops earlier in chikungunya patients (18,19). Nonetheless, until arthralgia, rash, or other clinical signs or symptoms have been shown to clearly differentiate patients with chikungunya from those with dengue, the introduction of chikungunya to the Americas heightens the clinical challenge of ensuring optimal management of dengue patients†† (i.e., hospitalization of patients with warning signs of severe dengue [e.g., persistent vomiting, severe abdominal pain] or other severe manifestations, and providing outpatients with appropriate anticipatory guidance). Because persons with suspected chikungunya might have dengue, nonsteroidal anti-inflammatory drugs (e.g., aspirin and ibuprofen) should be avoided and fever and pain should be managed with acetaminophen until there is a clear diagnosis and the patient is free of warning signs.

Although severe disease manifestations were uncommon among CHIKV-infected persons in Puerto Rico, clinicians should be aware of severe manifestations that have been previously associated with CHIKV infection (e.g., encephalitis and vesiculobullous skin lesions). Future investigations should describe the incidence and clinical course of patients in the Americas with severe manifestations of CHIKV infection, and verify previously identified risk factors for developing them (e.g., hypertension, underlying respiratory or cardiac conditions, and age ≥40 years) (15). Similarly, additional investigation is needed to determine the relative contribution of CHIKV infection and underlying medical conditions in the four fatal cases thus far reported. Finally, because CHIKV infection has been associated with persistent joint pain for months after the initial illness (14), additional investigation in the Americas is needed to quantitate disability caused by chikungunya-associated persistent joint pain.

The findings in this report are subject to at least two limitations. First, because of the large volume of cases reported to PRDH, not all specimens were tested. Instead, priority was assigned to specimens from hospitalized patients and municipalities that had not yet identified a laboratory-positive case. Thus, age- and municipality-specific incidence of chikungunya could not be accurately calculated. Second, household investigations were conducted during the daytime on weekdays, when children and working-age adults might not be available. Older persons might spend more hours at home during the day, when *Ae. aegypti* mosquitoes are most active, and might also have preexisting arthritis or other health conditions that might be exacerbated following CHIKV infection. Consequently, the rates of CHIKV infection and the associated symptoms identified through household investigations might not be representative of the population.

Because of the high volume of reported chikungunya cases, a network of sentinel chikungunya surveillance sites was established across Puerto Rico to better monitor the progression and trends of the chikungunya epidemic and the concurrent incidence of dengue. Clinical education seminars have been conducted throughout Puerto Rico to improve provider awareness of chikungunya, including the need to manage patients with suspected chikungunya the same way that dengue patients are managed. Messages to the public have emphasized the need to dispose of or empty water containers that can serve as mosquito breeding sites (e.g., refuse, discarded tires, and flower pots), and recommended seeking care early for acute febrile illness and managing fever and pain with acetaminophen. The chikungunya epidemic is expected to continue until a critical proportion of the population is no longer susceptible to infection. Forecasting the duration of the epidemic might be achieved through serologic surveys to monitor increases in population immunity.

Residents of and travelers to areas of the tropics with ongoing CHIKV and DENV transmission should employ mosquito avoidance strategies to prevent illness. Such strategies should include use of mosquito repellent, wearing long sleeves and pants, and staying in residences with air conditioning and screens on doors and windows. Additional information on chikungunya, including up-to-date case counts and affected areas, is available at www.cdc.gov/chikungunya.

## Figures and Tables

**TABLE:** Characteristics and signs and symptoms of chikungunya cases reported to the Puerto Rico Department of Health or detected through household investigations — Puerto Rico, 2014

	Reported cases (N = 652)		Recently ill household investigation participants (N = 59)	
		
Characteristic	No.	(%)	No.	(%)
**Demographics and clinical course**				
History of recent travel*	31	(5)	1	(1)
Female	344	(53)	27	(46)
Pregnant	8	(1)	1	(2)
Hospitalized	84	(13)	10	(17)
Median days from onset to specimen collection (range)	1 (0–40)		18 (1–60)	
**Signs and symptoms** †				
Fever	567	(87)	55	(93)
Arthralgia	512	(79)	56	(95)
Myalgia	518	(79)	48	(81)
Headache	434	(67)	41	(69)
Chills	343	(53)	42	(71)
Rash	263	(40)	32	(54)
Eye pain	282	(43)	18	(31)
Nausea/Vomiting	170	(26)	19	(32)
Abdominal pain	117	(18)	16	(27)
Arthritis§	97	(15)	29	(49)
Nasal congestion	87	(13)	12	(20)
Cough	92	(14)	13	(22)
Sore throat	101	(15)	14	(24)
Diarrhea	74	(11)	17	(29)
Conjunctivitis	16	(2)	20	(34)
Bleeding manifestations	175	(27)	7	(12)
Minor¶	172	(26)	7	(12)
Major**	17	(3)	0	(0)
Severe manifestations††	16	(2)	0	(0)

* Travel outside of Puerto Rico and the United States in the 14 days before illness onset.

† Signs and symptoms were either defined by a clinician for reported cases or were self-reported by household investigation participants.

§ Five cases reported arthritis in the absence of arthralgia. No investigation participants reported arthritis in the absence of arthralgia.

¶ Petechiae, bleeding gums, epistaxis, unspecified mucosal bleeding, and hematuria.

** Purpura/ecchymosis (16 persons), melena (two), hematemesis (two), and vaginal bleeding (one).

†† Jaundice (nine persons), convulsions (four), effusion (two), encephalitis (one), and hepatomegaly (one).

## Footnotes

* Additional information available at http://www.cdc.gov/chikungunya.

† Up-to-date case counts available at http://www.paho.org/hq/index.php?itemid=40931.

§ Additional information available at http://www.cdc.gov/dengue.

¶ Additional information available at http://www.healthmap.org/dengue/en.

** Available at http://www.cdc.gov/dengue/resources/denguecasereports/dcif_english.pdf.

†† Dengue clinical case management online training course available at http://www.cdc.gov/dengue/training/cme.html.
